# There is an excess of surgical site infections in Argentina

**DOI:** 10.1186/2047-2994-4-S1-O27

**Published:** 2015-06-16

**Authors:** RE Quirós

**Affiliations:** 1 Prevention and Infection Control Department, Hospital Universitario Austral, Pilar, Argentina

## Introduction

Since 2003 three Surgical Site Infection (SSI) surveillance systems were consecutive implemented in our country (IRIQ Project, 2003; VALIDAR Project, 2004; VIHDA System, 2005-2013) using standard methodology (NNISs, 1992-2004; NHSN 2006-2008). These systems have generated a local benchmark for Argentinean hospitals. However, it would be appropriate to compare local data with those international standards, in order to determine if there is, in our country, an excess in the number of SSI adjusted by surgical category and risk index.

## Objectives

To estimate the excess of SSI using the Standardized Infection Ratio (SIR) from three local surveillance systems compared with two external standards.

## Methods

Through a retrospective analysis of data from three Argentinean surveillance systems, 16 surgical procedures were selected according to the relevance of SSI associated and the lower probability of underreporting because the lack of post-discharge surveillance. Data from each local surveillance system were compared with external standard for the same time period through SIR. SSI excess was estimated as the difference between observed and expected cases. Between Oct-Dec 2014, an anonymous survey was sent to 165 hospitals, participating in at least one local surveillance system, with the purpose to evaluate the compliance with recommended strategies (SHEA-IDSA, 2014 Update) to prevent SSI.

## Results

While, for IRIQ Project, 3,186 surgeries were evaluated with an excess of 88 SSI (SIR 2.21; 95% CI 1.87–2.55; p<0.01), for VALIDAR Project, 5,762 procedures were included with an excess of 89 SSI (SIR 1.66; 95% CI 1.44–1.88; p<0.01), and for VIHDA System, 27,389 surgeries were evaluated with an excess of 462 SSI (SIR 1.91; 95% CI 1.79–2.03; p<0.01). While 68.8% of the procedures, showed a SIR significantly >1, none of the rest categories reach a SIR significantly <1. Among the surgical procedures with greater SIR were: cardiac surgery (coronary and non-coronary), craniotomy, spinal fusion, and hip and knee replacement. From the 82 centers (49.7%) who responded the survey, more than 50% failed in maintain a level of compliance above 90% for the preventive measures.

## Conclusion

This study shows that in our country there is a significant excess of SSI, even after adjusting for surgical category and risk index. This deviation could be associated with failures in the implementation of recommended preventive strategies.

## Disclosure of interest

None declared.

